# The pCONUS HPC: 30-Day and 180-Day In Vivo Biocompatibility Results

**DOI:** 10.1007/s00270-019-02202-z

**Published:** 2019-03-13

**Authors:** Pervinder Bhogal, Tim Lenz-Habijan, Catrin Bannewitz, Ralf Hannes, Hermann Monstadt, Andreas Simgen, Ruben Mühl-Benninghaus, Wolfgang Reith, Hans Henkes

**Affiliations:** 1 Department of Interventional Neuroradiology, The Royal London Hospital, Whitechapel Road, London, E1 1BB UK; 2 phenox GmbH, Bochum, Germany; 3 Neuroradiologische Klinik, Neurozentrum, Klinikum Stuttgart, Stuttgart, Germany; 4 Medical Faculty, University Duisburg-Essen, Essen, Germany; 5 Department of Neuroradiology, Saarland University, Homburg, Germany

**Keywords:** pCONUS, Stent, Wide-neck aneurysm, pHPC

## Abstract

**Background:**

Endovascular stents are commonly used during neurointerventional procedures; however, the concomitant use of dual anti-platelet treatment (DAPT) can limit their use. There is a need to develop stent coatings that mitigate requirement for DAPT.

**Methods:**

The hydrophilic polymer coating is a novel glycan-based multilayer polymer that inhibits platelet adhesion. After Institutional Animal Care and Use Committee approval, 18 New Zealand white rabbits (mean weight 4.02 ± 0.51 kg) were commenced on DAPT (ASA 10 mg/kg/day and clopidogrel 10 mg/kg/day). A bare nitinol pCONUS and coated pCONUS HPC were implanted into the common carotid arteries of each rabbit. Histological examinations were performed at 30 days (*n* = 9) and 180 days (*n* = 8) to assess the acute and chronic inflammatory reactions to the pCONUS HPC. Wilcoxon/Kruskal–Wallis and ANOVA were used with *p* value < 0.05 considered as significant.

**Results:**

There is no statistically significant difference in inflammation within the intima/media or adventitia at 30 days (*p* = 0.3901 and *p* = 1, respectively) or at 180 days (*p* = 0.144 and *p* = 1, respectively) between pCONUS and pCONUS HPC cohorts. There is no significant difference in the presence of granulomas or giant cells between the cohorts at either 30 days (*p* = 1 and *p* = 0.8363) or 180 days (*p* = 1.00 and *p* = 0.149). At 30 days and 180 days, there was near-complete endothelialisation of the stent struts and no significant difference between the pCONUS or pCONUS HPC (*p* = 0.7832 and *p* = 0.334, respectively).

**Conclusion:**

pCONUS HPC stents do not elicit an acute or chronic inflammatory response in vivo with no significant difference in the tissue response to bare nitinol pCONUS stents or pCONUS HPC stents.

## Introduction

Wide-neck bifurcation aneurysms (WBNA’s) are a challenging entity to treat, and a wide variety of different adjunctive methods have been reported in the literature [1–7]. Almost two-thirds of all intracranial aneurysms occur at bifurcations [8] with wide-neck aneurysms, typically defined as those with maximal neck dimension ≥ 4 mm and/or aspect ratio < 1.4, posing a particular challenge for endovascular treatment [9, 10]. A variety of techniques have been developed to deal with difficult aneurysmal morphology including balloon remodelling, waffle-cone stenting, Y-stenting and T-stenting. Recently, numerous dedicated devices have entered the market which have been designed to tackle this difficult entity and they include the pCONUS 1, pCONUS 2 [11], pCANvas [12] (Phenox, Bochum, Germany), the PulseRider [13] (Pulsar Vascular, Los Gatos, California, USA), the Barrel vascular reconstruction device (Medtronic, Dublin, Ireland) and the eCLIPs device [14] (Evasc Medical Systems Corp.). These devices share the common feature of providing extra coverage at the aneurysm neck to prevent coil prolapse into the parent vessel.

One problem of both the aneurysm remodelling techniques, such as T-stenting, and specifically designed stent-like devices is the requirement for adequate anti-platelet medication to prevent thrombosis of the stent, which can cause problems particularly in the acute scenario. The use of stents or stent-like structures such as the pCONUS in the acute situation is thought to increase the risk of bleeding-related complications if interventions such as the insertion of an EVD (extra-ventricular drain) are required [15–20]. This increased risk is believed to be principally due to the requirement for concomitant anti-platelet medication. Bodily et al. [21] performed a systematic review of the literature to analyse the risks associated with the use of stents with acutely ruptured intracranial aneurysms. They identified 17 articles and 339 patients and a total haemorrhagic complication rate of 8% (27/339); however, 1/3 (*n* = 9) of these patients were haemorrhage related to the EVD and nearly half (*n* = 12) were intra-procedural rupture of the aneurysm. Similarly, they reported clinically significant thromboembolic events in 6% of cases with available data. In the publication of Kung et al. [22], symptomatic and radiographic haemorrhage was seen in 32% of cases for patients on DAPT compared to 14.7% for those not on anti-platelet medications. Although the pCONUS device has been used in the acute setting [23], it is not without risk and there is a need to develop strategies to allow the use of adjunctive devices whilst minimising the risk of both haemorrhagic and thromboembolic complications. One avenue is the development of devices that will not require dual anti-platelet treatment (DAPT). Such devices are now entering clinical use although there are only sparse data available at the current time with regards to their safety and efficacy [24, 25].

We have recently shown that the hydrophilic polymer coating (pHPC, registered trademark of Phenox) has anti-thrombogenic properties when tested in vitro [26], but there is little known about the in vivo biocompatibility of this coating. The purpose of this study was to determine the acute and chronic biocompatibility of the pHPC coating when applied to the pCONUS device.

## Materials and Methods

### Animal Experiments and Pre-medication

After Institutional Animal Care and Use Committee approval, 18 female New Zealand white rabbits, mean average weight 4.02 ± 0.51 kg, and of similar age, were commenced on DAPT with acetylsalicylic acid (ASA) and clopidogrel (ASA 10 mg/kg/day and clopidogrel 10 mg/kg/day). The medication was commenced 72 h prior to the planned intervention and continued for the duration of the study. The medication was provided to the animals via their drinking water. Ten rabbits were assigned to the 30-day cohort, and eight rabbits were assigned to the 180-day cohort.

The rabbit model was chosen as the supra-aortic vessels having an appropriate diameter for clinically available pCONUS and pCONUS HPC stents. Furthermore, the rabbit model is the most extensively used model to assess neurovascular devices prior to human use [27].

### Stent Implant Procedure

All procedures were performed with the animals under general anaesthesia with Ketamin (60 mg/kg)/Rompun 2% (6 mg/kg) IM and maintenance with Ketamin (60 mg/kg)/Rompun 2% (6 mg/kg) in 10 ml NaCl at a flowrate of 2.5 ml/h via the ear vein. The right common femoral artery was surgically exposed and a 4Fr introducer sheath inserted. Using a 4Fr vertebral catheter (Glidecath, Terumo Europe, Leuven, Belgium), angiography of the common carotid arteries (CCA) was performed. After full heparinisation and activated clotting time (ACT) 2–2.5 times normal, a 0.021 inch Trevo Pro 18 microcatheter (Stryker, Kalamazoo, USA) or Prowler Select plus (Codman Neurovascular, West Chester, USA) with 0.014inch pORTal microwire (Phenox, Bochum, Germany) were used to access the CCA’s and for deployment of the pCONUS device.

### Stent Characteristics

The pCONUS is a CE marked device and is available for clinical use in Europe. The device is an electrolytically detached laser cut nitinol stent with a distal crown consisting of four ‘petals’ that can unfold. These petals are deployed within the aneurysm dome and positioned at the base of the aneurysms in order to provide neck coverage for wide-neck aneurysms and to assist with coiling. The device is available in a wide range of sizes.

### Implant Location and Stent Sizing

In each animal, the pCONUS was implanted into the left common carotid artery (CCA) and the pCONUS HPC was implanted into the right CCA. The CCA was chosen given its similar size to the intracranial arteries of humans.

In all animals, the same size pCONUS and pCONUS HPC device (3 × 20 × 4 mm) were implanted.

### pHPC Surface Coating

The initial results of in vitro testing of the pHPC coating were recently published [26], and in brief, these demonstrated that the coating could be successfully applied to nitinol. The thickness of the surface coatings is between 10 and 20 nm as determined by XPS analysis. The thin nature of the coating has influence nether neither on the surface texture nor on the physical properties of the stents. The coating itself is a newly developed glycan-based multilayer polymer.

In vitro testing showed a significant reduction in the adherence of immunofluorescent CD61^+^ platelets when incubated with whole blood from healthy volunteers compared to uncoated stents. Scanning electron microscopy also demonstrated minimal adherent platelets on the coated flow diverters compared to a thick layer of adherent platelets on uncoated stents.

### Angiographic Imaging

Control angiography was performed following implantation of the pCONUS device and 30 or 180 days after implantation. Selective DSA at final follow-up was performed via the left common femoral artery using a 4Fr vertebral catheter (Glidecath, Terumo Europe, Leuven, Belgium) after surgical exposure and introduction of a 4Fr introducer sheath.

### Harvest and Gross Imaging

Euthanasia by pentobarbital (Narcoren, Merial GmbH, Hallbergmoos, Germany) overdose (6–8 ml) was performed at 30 or 180 days, whilst the animals were under anaesthesia with Ketamin (60 mg/kg)/Rompun 2% (6 mg/kg) in 10 ml NaCl at a flowrate of 2.5 ml/h via the ear vein.

**Fig. 1:**
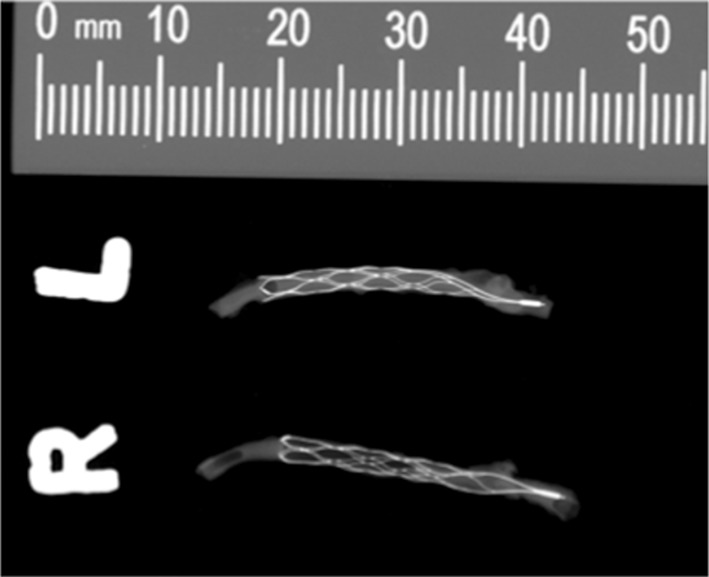
Radiographs of the resected devices from animal 5 demonstrating good wall apposition of both the pCONUS and pCONUS HPC devices

The arterial segments with the implanted pCONUS’s and pCONUS HPC’s were surgically removed and fixed in 10% formaldehyde. All specimens were photographed and radiographed using a LX-60 cabinet radiography system (Faxitron, Arizona, USA) (Fig. 1).

### Histological Preparation

After gross imaging, the excised arterial segments were dehydrated in a graded series of ethanol and embedded in Spurr’s epoxy resin. After polymerisation, transverse section from the proximal, middle and distal segments of the pCONUS shaft was taken and the cross sections adhered to plastic slides. The slides were prepared to a thickness of 32–88 microns (Exakt, Oklahoma City, USA). The slides were then polished and stained with haematoxylin and eosin (H&E) stain.

### Histological and Morphological Assessment

Morphometric analysis was performed on each segment by an independent, experienced observer (CV Path, Gaithersburg, MD, USA) using digital planimetry with a calibrated camera. For each prepared section, a morphometric analysis was performed and included the luminal area of the vessel, the area of the internal and external elastic laminae (IEL and EEL, respectively) and the neointimal thickness that was calculated as the distance from the IEL to luminal border. Semi-quantitative data including medial fibrin and calcification deposition, medial and adventitial inflammatory cell invasion and adventitial thickness were recorded (Table 1). Histopathological analysis was conducted by an independent pathologist (CV Path, Gaithersburg, MD, USA) blinded to the coating status of the pCONUS.

**Table 1:** Description of semi-quantitative histology scores

Scored parameter	0 (none)	1 (minimal)	2 (mild)	3 (moderate)	4 (severe)
*Tissue matrix*					
Fibrin (media)	none	Minimal, focal	Mild, multifocal	Moderate, regionally diffuse	Severe, marked diffuse
Calcification	none	Focal with < 10% of the region affected	Multifocal with 10–25% of the region affected	Regionally diffuse with 26–30% of the region affected	Regionally diffuse with > 30% of the region affected
*Inflammation*					
Inflammation (intima/media)	none	< 20 inflammatory cells/HPF in < 25% of area	21–100 inflammatory cells/HPF in 25–50% of area	101–150 inflammatory cells/HPF > 51–75% of area	> 150 inflammatory cells/HPF > 75% of area
Inflammation (adventitia)	none	< 20 inflammatory cells/HPF in < 25% of area	21–100 inflammatory cells/HPF in 25–50% of area	101–150 inflammatory cells/HPF > 51–75% of area	> 150 inflammatory cells/HPF > 75% of area
*Adventitial fibrosis*					
Adventitial thickening	none	< 25% of the area	25–50%	51–75%	> 75%

Slides were stained with haematoxylin and eosin, and all sections were examined by light microscopy (20× as the maximum magnification). The presence of giant cells and granulomas on the stent struts was assessed and calculated as a percentage.

The media area (EEL area–IEL area), neointimal area (IEL-luminal are) and per cent luminal stenosis (1 − [luminal area/IEL area] × 100) were also calculated.

## Statistics

For morphometric measurements, an ANOVA was used for unpaired comparisons to calculate the significance of differences between the cumulative frequency distribution of coating groups. Semi-quantitative (ordinal) data including surface platelet/fibrin deposition, injury, medial smooth muscle cell loss, neointimal/medial, and adventitia inflammation, medial and adventitial haemorrhage and fibrin (Table 1) were compared using the nonparametric Wilcoxon/Kruskal–Wallis (Rank Sums) test. A value of *p* ≤ 0.05 was considered statistically significant.

## Results

### 30-day Results

Nine of the ten rabbits in the 30-day cohort survived until the end of the experiment. In two of the rabbits, the right CCA was occluded, and therefore, a pCONUS HPC was not implanted; therefore, in total, seven pCONUS HPC and nine pCONUS devices were implanted and available for analysis at 30-days.

#### Morphological and Histological Analysis

The morphometric findings were similar between pCONUS and pCONUS HPC devices. There was no statistically significant difference between the luminal area of the vessel, the area of the IEL, EEL or the neointimal thickness. Similarly, there was no evidence of greater stenosis of the vessel between the pCONUS and pCONUS HPC cohorts. The results are summarised in Table 2.

**Table 2:** Morphometric comparison of cross-sectional vessel areas and neointimal thickness (values represent the mean ± SD of proximal, middle and distal sections) and histologic comparison of vessel injury and healing of pCONUS HPC and pCONUS stent implants in the carotid arteries of healthy rabbits that survived for 30-days

Implant group		EEL area (mm^2^)	IEL area (mm^2^)	Lumen area (mm^2^)	Medial area (mm^2^)	Neointimal area (mm^2^)	Stenosis (%)	Neointimal thickness (mm)	Tissue matrix		Inflammation				Neoendothelialisation
									Fibrin (media)	Calcification	Intima/media inflammation	Adventita inflammation	Struts with granulomas (%)	Struts with giant cells (%)	Uncovered struts
pCONUS HPC	*n* = 7	2.49 ± 0.56	2.14 ± 0.50	1.92 ± 0.47	0.35 ± 0.07	0.21 ± 0.06	10.16 ± 2.18	0.06 ± 0.02	0.00 ± 0.00	0.00 ± 0.00	0.10 ± 0.16	0.00 ± 0.00	0.00 ± 0.00	9.96 ± 14.04	0.60 ± 1.57
pCONUS	*n* = 9	2.55 ± 0.63	2.20 ± 0.58	2.00 ± 0.55	0.35 ± 0.05	0.21 ± 0.07	9.50 ± 2.83	0.06 ± 0.03	0.00 ± 0.00	0.00 ± 0.00	0.04 ± 0.11	0.00 ± 0.00	0.00 ± 0.00	8.80 ± 7.92	0.93 ± 2.78
*p* value		0.8319	0.815	0.7875	0.9919	0.8569	0.6203	0.7999	1	1	0.3901	1	1	0.8363	0.7832

**Fig. 2:**
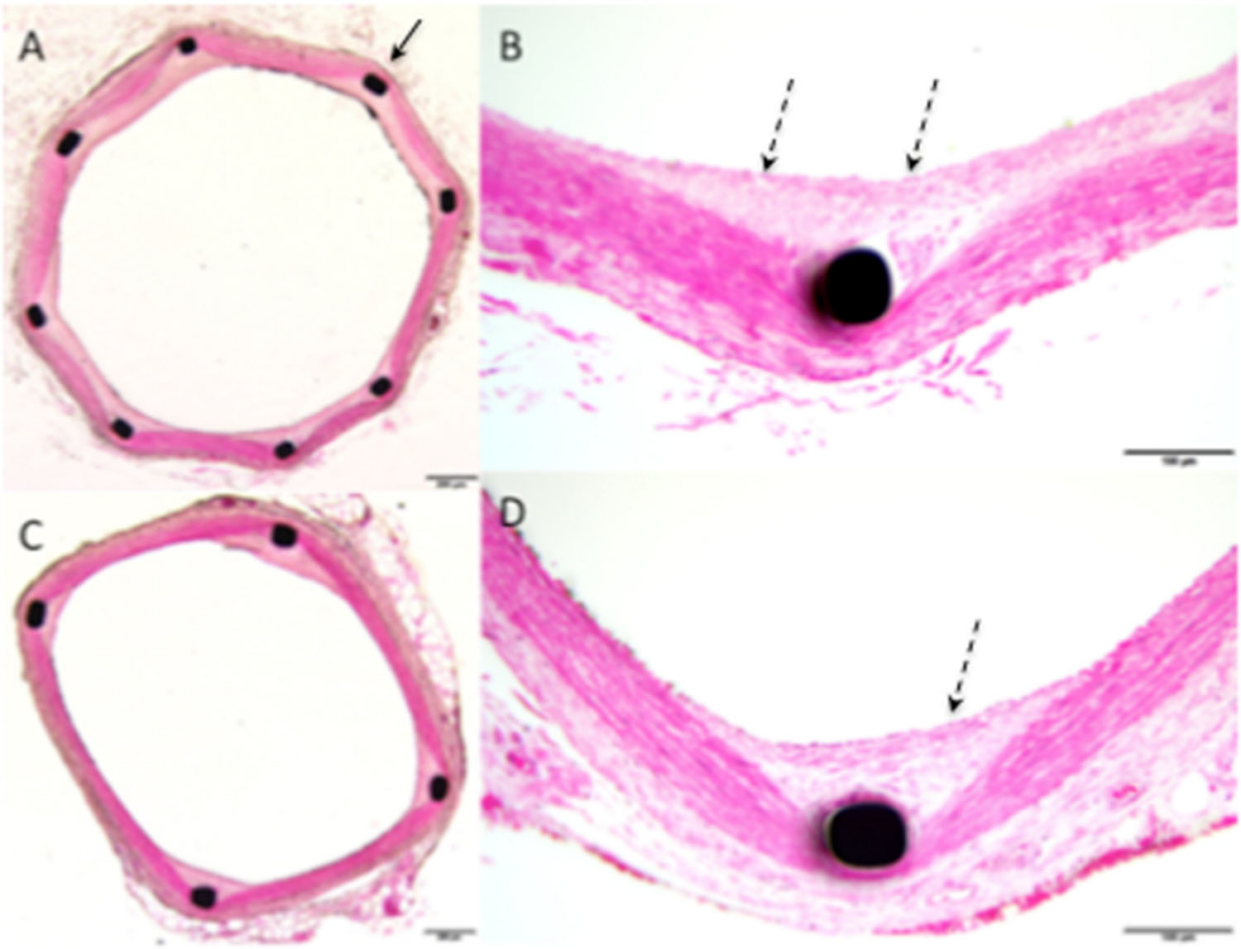
Histological imaging of animal 1, performed at 30 days post-implantation, showed smooth muscle cell (SMC) rich neointima (dashed black arrows) covering the struts (solid black arrow) of the pCONUS for both the uncoated (**A**, **B**) and coated pCONUS HPC (**C**, **D**). At high power magnification (×20) there was no evidence of significant inflammatory cell invasion (**B**, **D**). There was no evidence of calcification

**Fig. 3:**
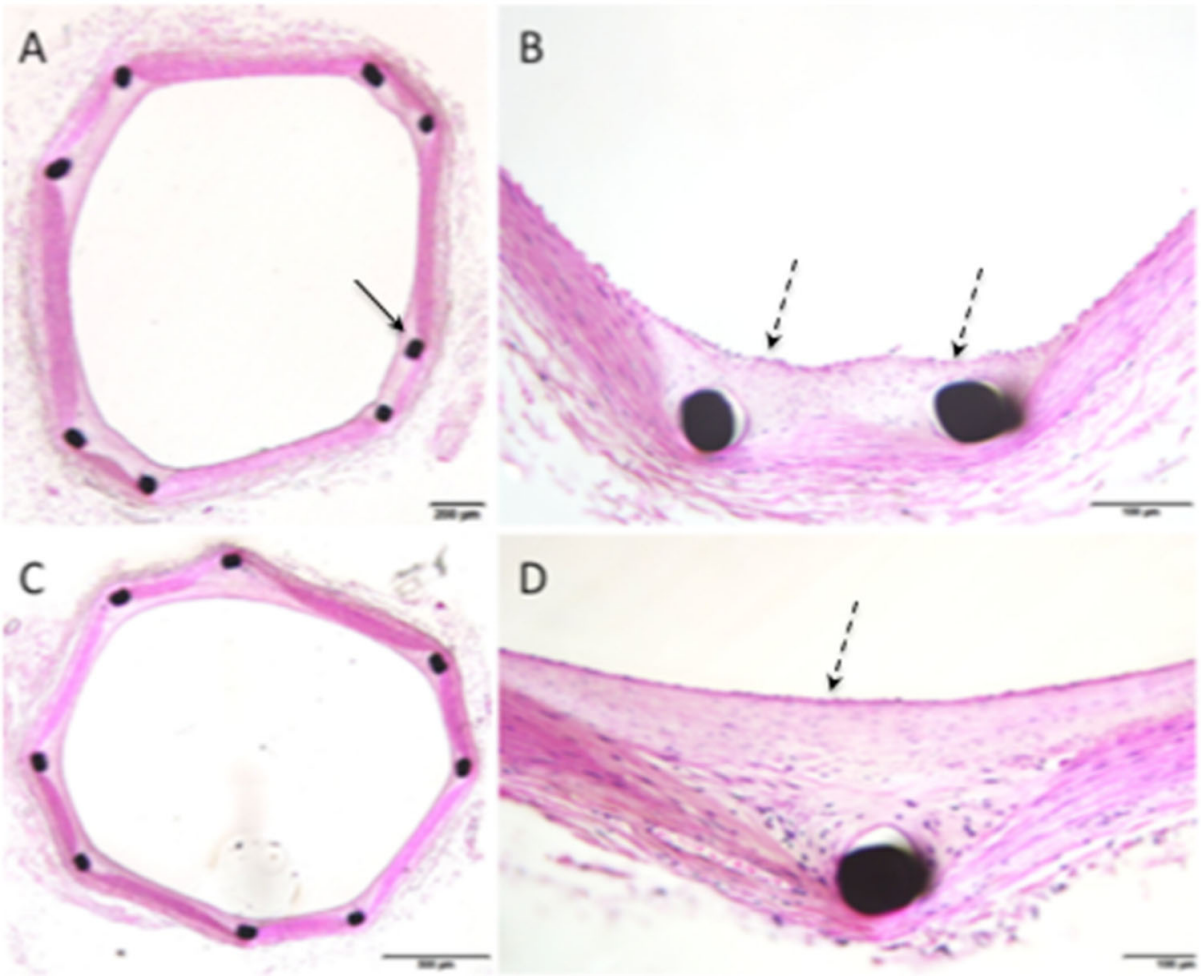
Histological imaging of animal 11, performed at 180 days post-implantation, showed SMC rich neointima (dashed black arrows) completely covering the struts (solid black arrow) of the pCONUS (**A**, **B**) and pCONUS HPC (**C**, **D**) with no evidence of chronic inflammation. There was no evidence of adventitial inflammation

There was no evidence of significantly greater inflammation within the intima, media or adventitial arterial wall components between the cohorts (Figs. 2, 3). Importantly, there was no evidence of granuloma formation in either cohort and no statistical difference in the percentage of stent struts harbouring giant cells (*p* = 0.8363). Both stents appeared to be virtually completely covered in neoendothelium with less than 1% of the stent struts being nonendothelialised at 30-days (0.6 ± 1.57 vs. 0.932.78, *p* = 0.7832). The results are summarised in Table 2.

### 180-day Results

#### Morphological and Histological Analysis

Overall morphometric findings were similar between pCONUS and pCONUS HPC devices. There was no statistically significant difference between the luminal area of the vessel, the area of the IEL, EEL or the neointimal thickness. Similarly, there was no evidence of greater stenosis of the vessel between the pCONUS and pCONUS HPC cohorts. Stenosis values were minimal and slightly higher in the uncoated controls (9.60 ± 4.01 vs. 12.06 ± 4.30, *p* = 0.255). The results are summarised in Table 3.

**Table 3:** Morphometric comparison of cross-sectional vessel areas and neointimal thickness (values represent the mean ± SD of proximal, middle and distal sections) and histologic comparison of vessel injury and healing of pCONUS HPC and pCONUS stent implants in the carotid arteries of healthy rabbits that survived for 180-days

Implant group		EEL area (mm^2^)	IEL area (mm^2^)	Lumen area (mm^2^)	Medial area (mm^2^)	Neointimal area (mm^2^)	Stenosis (%)	Neointimal thickness (mm)	Tissue matrix		Inflammation				Neoendothelialisation
									Fibrin (media)	Calcification	Uncovered struts	Adventita inflammation	Struts with granulomas (%)	Struts with giant cells (%)	Uncovered struts
pCONUS HPC	*n* = 8	2.16 ± 0.41	1.85 ± 0.41	1.69 ± 0.41	0.30 ± 0.022	0.17 ± 0.047	9.60 ± 4.01	0.036 ± 0.018	0.00 ± 0.00	0.00 ± 0.00	0.00 ± 0.00	0.00 ± 0.00	0.00 ± 0.00	0.00 ± 0.00	2.08 ± 5.89
pCONUS	*n* = 8	1.94 ± 0.39	1.65 ± 0.37	1.46 ± 0.37	0.30 ± 0.033	0.19 ± 0.062	12.06 ± 4.30	0.048 ± 0.019	0.042 ± 0.12	0.00 ± 0.00	0.13 ± 0.25	0.00 ± 0.00	0.00 ± 0.00	2.08 ± 3.86	0.00 ± 0.00
*p* value		0.309	0.317	0.267	0.543	0.419	0.255	0.226	0.317	1	0.144	1	1	0.149	0.334

On histological analysis, there was no evidence of significant medial fibrin deposition or calcification (Figs. 2, 3). There was no significant inflammation within either the intima, media or adventitial arterial wall components and again no significant difference in the presence of granulomas (*p* = 1.00) or giant cells (*p* = 0.149) on the stent struts. There was no adventitial reaction observed for either test or control cohort. There was virtually complete neoendothelialisation for both cohorts with no significant difference between the percentage of uncovered stent struts (*p* = 0.334). The results are summarised in Table 3.

## Discussion

The results of this in vivo study demonstrate that the pCONUS HPC does not elicit a statistically significant inflammatory response, acute or chronic, greater than an uncoated pCONUS. Similarly, there is no evidence of a fibrotic reaction within the arterial wall as a response to chronic long-term exposure to the pCONUS HPC nor is there any evidence of a difference in neoendothelialisation between the pCONUS and pCONUS HPC. This study was designed to determine if the pCONUS HPC elicited an acute or chronic inflammatory response in the arterial wall. No other benefits of the HPC coating, for example, the efficacy of the pCONUS HPC under single anti-platelet treatment (SAPT), can be gleaned from this study. However, these results, taken together with our previously published in vitro [26] and in vivo [28] results, suggest that the pHPC coating has strong anti-thrombogenic properties and does not promote an inflammatory reaction.

The cellular responses that occur following mechanical vascular injury immediately after stent deployment is tri-phasic [12–14]:

1. Early phase—platelet activation and inflammation
2. Intermediate phase—granulation tissue and smooth muscle cell migration and proliferation
3. Late phase—tissue remodelling

In the early phase, endothelial cells are partially/completely destroyed or crushed resulting in platelet activation and aggregation, leucocyte infiltration and the release of growth factors and cytokines [12, 15, 16]. The initial endothelial damage initiates the formation of a thin thrombus layer that covers the vascular and stent surface even in the presence of DAPT’s and heparin [13]. Following platelet activation and aggregation, there is recruitment of circulating leucocytes [16] and this leucocyte-platelet interaction is crucial in the neointimal regeneration process [17].

During the intermediate phase, endothelial cells proliferate and migrate over the injured areas with vascular smooth muscle cells (VSMC’s) and macrophages replacing the fibrin clot with granulation tissue. During the late tissue-remodelling phase, the VSMC’s undergo a phenotypic switch from contractile to synthetic VSMC’s and this is important for the generation of extracellular matrix (ECM), which is eventually deposited in the intima [18]. Eventually, a permanent matrix of type 1 and 3 collagen is produced that completes the healing process. The results of our study suggest that this process of repair is not inhibited by the pCONUS HPC as there was no significant difference in the neoendothelialisation at either the 30-day or 180-day time points.

Although the pCONUS has been used in the acute setting it is not without risk [23]. Our previous work has shown the HPC coating to have strong anti-thrombogenic properties and in unison with the current results suggest that the pCONUS HPC may be suitable for use with mono-aggregation.

We recognise that this study and the conclusions may have limitations and the translation into clinical practice needs to be carefully considered. The follow-up period of the study was just 6 months, and therefore, the effect of the device coating when implanted for even longer time periods is not known. Similarly, the pCONUS is used to assist with the coiling of aneurysms and therefore the interaction of the pCONUS HPC with different coils is unknown. It is also important to note that in this study DAPT was used and it is possible that under single anti-platelet treatment the results may differ. Furthermore, a limited number of subjects were used and larger cohorts are necessary.

## Conclusion

The pCONUS HPC stents do not elicit either an acute or chronic inflammatory response in vivo, and there is no significant difference between the tissue response to bare nitinol pCONUS stents and pCONUS HPC stents.
